# Predictors of acute genitourinary toxicity in real-time image-gated spot-scanning proton beam therapy for prostate cancer

**DOI:** 10.1093/jrr/rrag012

**Published:** 2026-03-16

**Authors:** Sho Seri, Norihiro Aibe, Takuya Kimoto, Toshiyuki Ogata, Kanako Kawabata, Koji Masui, Shinsuke Nagasawa, Yuki Yoshino, Sho Watanabe, Akito Asato, Shoko Hirano, Satoshi Ikeda, Takumi Shiraishi, Atsuko Fujihara, Hideya Yamazaki, Osamu Ukimura, Kei Yamada, Gen Suzuki

**Affiliations:** Department of Radiology, Kyoto Prefectural University of Medicine, 465 Kajiicho, Kawaramachi-Hirokoji, Kamigyo-ku, Kyoto 602-8566, Japan; Department of Radiology, Kyoto Prefectural University of Medicine, 465 Kajiicho, Kawaramachi-Hirokoji, Kamigyo-ku, Kyoto 602-8566, Japan; Department of Radiology, Kyoto Prefectural University of Medicine, 465 Kajiicho, Kawaramachi-Hirokoji, Kamigyo-ku, Kyoto 602-8566, Japan; Department of Radiology, Kyoto Prefectural University of Medicine, 465 Kajiicho, Kawaramachi-Hirokoji, Kamigyo-ku, Kyoto 602-8566, Japan; Department of Radiology, Kyoto Prefectural University of Medicine, 465 Kajiicho, Kawaramachi-Hirokoji, Kamigyo-ku, Kyoto 602-8566, Japan; Department of Radiology, Kyoto Prefectural University of Medicine, 465 Kajiicho, Kawaramachi-Hirokoji, Kamigyo-ku, Kyoto 602-8566, Japan; Department of Radiology, Kyoto Prefectural University of Medicine, 465 Kajiicho, Kawaramachi-Hirokoji, Kamigyo-ku, Kyoto 602-8566, Japan; Department of Radiology, Kyoto Prefectural University of Medicine, 465 Kajiicho, Kawaramachi-Hirokoji, Kamigyo-ku, Kyoto 602-8566, Japan; Department of Radiation Oncology and Kansai BNCT Medical Center, Osaka Medical and Pharmaceutical University, 2-7 Daigaku-machi, Takatsuki, Osaka 569-8686, Japan; Department of Radiology, Fukuchiyama City Hospital, 231 Atsunaka-cho, Fukuchiyama, Kyoto 620-8505, Japan; Department of Radiology, Kyoto Prefectural University of Medicine, 465 Kajiicho, Kawaramachi-Hirokoji, Kamigyo-ku, Kyoto 602-8566, Japan; Department of Radiology, Kyoto Prefectural University of Medicine, 465 Kajiicho, Kawaramachi-Hirokoji, Kamigyo-ku, Kyoto 602-8566, Japan; Department of Radiology, Kyoto Prefectural University of Medicine, 465 Kajiicho, Kawaramachi-Hirokoji, Kamigyo-ku, Kyoto 602-8566, Japan; Department of Urology, Graduate School of Medical Science, Kyoto Prefectural University of Medicine, 465 Kajiicho, Kawaramachi-Hirokoji, Kamigyo-ku, Kyoto 602-8566, Japan; Department of Urology, Graduate School of Medical Science, Kyoto Prefectural University of Medicine, 465 Kajiicho, Kawaramachi-Hirokoji, Kamigyo-ku, Kyoto 602-8566, Japan; Department of Radiology, Kyoto Prefectural University of Medicine, 465 Kajiicho, Kawaramachi-Hirokoji, Kamigyo-ku, Kyoto 602-8566, Japan; Department of Urology, Graduate School of Medical Science, Kyoto Prefectural University of Medicine, 465 Kajiicho, Kawaramachi-Hirokoji, Kamigyo-ku, Kyoto 602-8566, Japan; Department of Radiology, Kyoto Prefectural University of Medicine, 465 Kajiicho, Kawaramachi-Hirokoji, Kamigyo-ku, Kyoto 602-8566, Japan; Department of Radiology, Kyoto Prefectural University of Medicine, 465 Kajiicho, Kawaramachi-Hirokoji, Kamigyo-ku, Kyoto 602-8566, Japan

**Keywords:** real-time image-gated proton beam therapy, spot scanning, prognostic factors, acute adverse event, genitourinary toxicity, international prostate symptom score

## Abstract

Proton beam therapy (PBT) provides sharper dose gradients than conventional photon radiotherapy, potentially reducing radiation exposure to normal tissues. However, acute genitourinary adverse events (GU AEs) remain a clinical concern. This study aimed to evaluate the incidence and predictors of grade ≥ 2 acute GU AEs in patients with localized prostate cancer treated with real-time image-gated, spot-scanning PBT (RGPT). We analyzed the prospective study data of 326 patients who received RGPT at a dose of 63 Gy (relative biological effectiveness [RBE]) in 21 fractions between 2019 and 2021. Acute GU AEs were graded according to the Common Terminology Criteria for Adverse Events (version 5.0). Multivariable logistic regression was used to analyze potential predictive factors, including baseline International Prostate Symptom Score (IPSS), age, comorbidities, hydrogel spacer placement, use of anticoagulants or urinary symptom medications, clinical stage, National Comprehensive Cancer Network (NCCN) risk classification, prostate volume, and dose–volume histogram parameters. Grade 2 acute GU AEs occurred in 127 patients (39.0%), whereas no grade ≥ 3 events were observed. Moderate (odds ratio [OR] = 1.71; 95% confidence interval [CI]: 1.03–2.82) and severe (OR = 3.75; 95% CI: 1.49–9.46) baseline IPSS, as well as age ≥ 75 years (OR = 1.80; 95% CI: 1.10–2.95), were significant independent predictors of grade ≥ 2 GU AEs. No dose–volume histogram parameters were significantly associated with grade ≥ 2 GU AEs. Baseline urinary symptoms and older age were independent predictors of grade ≥ 2 GU AEs, emphasizing the importance of pretreatment evaluation in optimizing patient selection and management.

## INTRODUCTION

Prostate cancer is one of the most commonly diagnosed cancers worldwide, and its incidence in Japan has increased markedly with population aging, lifestyle westernization, and the widespread use of prostate-specific antigen screening. Growing health awareness and advances in diagnostic techniques have facilitated early detection of prostate cancer, underscoring the need to develop high-quality treatment options.

Radiation therapy is generally recognized for its ability to preserve patients’ quality of life (QOL), highlighting its importance as a treatment option for localized prostate cancer. Proton beam therapy (PBT) offers unique physical dose-distribution characteristics, particularly the Bragg peak phenomenon, in which protons deposit the majority of their energy at a specific depth, thereby minimizing radiation exposure to surrounding tissues [1, 2]. This facilitates a more precise dose delivery and may lead to better therapeutic outcomes [3]. Thus, PBT has gained increasing attention as a minimally invasive treatment option for prostate cancer [3, 4].

Several studies have reported that the therapeutic efficacy of hypofractionated PBT is comparable to that of conventionally fractionated PBT (2.0 Gy relative biological effectiveness [RBE]/fraction) [5, 6]. Thus, hypofractionated PBT is expected to become more popular, especially given its shorter treatment duration and reduced medical costs.

Alongside the increasing use of PBT, there is growing concern regarding genitourinary adverse events (GU AEs) during definitive radiation therapy for prostate cancer [7]. Such GU AEs can adversely affect QOL through symptoms such as urinary frequency, dysuria, and urgency, which often develop within 3 months of treatment [8]. Pretreatment urinary conditions have been suggested as potential risk factors for the development of these GU AEs [6, 9]. Accordingly, patient-reported outcome measures, such as the International Prostate Symptom Score (IPSS), have been widely implemented in clinical practice to assess patients’ health status [5, 10–12]. However, few published studies have examined the relationship between pretreatment IPSS and GU AEs following PBT for localized prostate cancer. Furthermore, the predictors of acute GU AEs in patients undergoing hypofractionated real-time image-guided spot-scanning proton beam therapy (RGPT) [12] remain insufficiently explored.

This study clarified the associations between pretreatment clinical factors and treatment-related factors with the incidence of acute GU AEs in patients receiving hypofractionated RGPT for prostate cancer. By identifying patients at higher risk of developing acute GU AEs, treatment options can be better personalized for each patient, with the potential to improve clinical outcomes and minimize adverse effects. To the best of our knowledge, this is the largest study to date investigating acute GU AEs in patients with prostate cancer treated with RGPT.

## MATERIALS AND METHODS

### Patient data

This prospective, single-institution study was approved by the Institutional Review Board of Kyoto Prefectural University of Medicine (IRB number: ERB-C-1499) and conducted in accordance with the Declaration of Helsinki. This analysis included patients who met the following eligibility criteria: (i) histologically confirmed prostate cancer with no prior treatment other than androgen deprivation therapy (ADT); (ii) no evidence of metastasis; (iii) treatment with RGPT at a dose of 63 Gy (RBE) in 21 fractions (5 fractions per week) between August 2019 and September 2021; (iv) treatment planning performed using a single computed tomography (CT) scan; (v) baseline IPSS assessment completed prior to RGPT; (vi) a minimum follow-up period of 3 months after completing PBT; and (vii) provision of written informed consent. Three patients (0.9%) had a history of prostate-related surgical procedures, such as transurethral resection of the prostate (TURP), which were not considered exclusion criteria. Patients requiring multiple CT scans for replanning were excluded due to the difficulty in accurately assessing the dose delivered to the target and organs at risk (OARs). The clinical diagnosis was made using TNM staging, and patients were classified according to NCCN risk categories [13]. Patients with intermediate- and high-risk disease generally received neoadjuvant ADT for at least 3 months prior to PBT, which is typically continued for up to 2 years in high-risk patients. Acute GU AEs were evaluated using the National Cancer Institute Common Terminology Criteria for Adverse Events (version 5.0) and were defined as events occurring within 90 days from the initiation of PBT.

### Treatment planning

For RGPT treatment planning, all patients underwent implantation of gold fiducial markers in the prostate prior to CT simulation [14, 15]. Eligibility for hydrogel spacer placement (SpaceOAR; Boston Scientific Corporation, Marlborough, MA, USA) [16], aimed at reducing rectal dose, was determined during a multidisciplinary institutional conference involving urologists, radiologists, and radiation oncologists. The primary criterion for spacer use was the absence of extracapsular dorsal tumor extension. Eligible patients were offered the procedure, which was performed in those who consented.

Patients were immobilized in the supine position using a customized vacuum cushion (Engineering Systems Corporation, Nagano, Japan). To ensure consistent bladder volume, all patients were instructed to void their bladder 60 min prior to each treatment session. Bladder filling was then assessed using pretreatment ultrasonography, and patients were instructed to consume an appropriate amount of water, based on the ultrasonographic findings, to maintain consistent bladder volume throughout the treatment course. Target volumes and OARs were delineated via CT scan (2.0-mm slice thickness) and T2-weighted magnetic resonance imaging (MRI). The clinical target volume (CTV) was defined based on institutional protocols in accordance with the NCCN risk classification [13].

Seminal vesicle (SV) involvement was assessed using pretreatment diagnostic MRI, with final determination of SV status made through a multidisciplinary cancer board consensus involving radiologists, urologists, and radiation oncologists. Furthermore, the ‘base’ of the SV was defined as the proximal portion of the SV, specifically the segment within 10 mm of the prostate–SV junction. For low-risk patients, the CTV included the prostate with a 3-mm margin. For intermediate-risk patients, the CTV included the prostate plus the SV base with a 3-mm margin. For high-risk patients without SV invasion, the CTV included the prostate and the proximal half of the seminal vesicles, both with a 3-mm margin. In patients with SV involvement, the CTV included both seminal vesicles with a 3-mm margin. The CTV was subsequently modified based on individual anatomical boundaries. The bladder and rectum were contoured as OARs according to the Pelvic Normal Tissue Contouring Guidelines for Radiation Therapy by the Radiation Therapy Oncology Group consensus panel atlas, with the rectum delineated following the AnoRectumSig [17]. The prescribed dose of 63 Gy (RBE) in 21 fractions was required to cover at least 98% of the CTV, and the treatment plan was designed to meet institutional dose constraints (Table 1). Treatment planning was performed using the VQA system (Hitachi, Ltd., Tokyo, Japan) with a single-field uniform-dose proton therapy approach using four beams.

**Table 1:** Dose constraints or organs at risk

	Goal	Minor variation	Major variation
Rectum			
V30 Gy (RBE) (%)	<30	<32.5	<35
V45 Gy (RBE) (%)	<15	<17.5	<20
V60 Gy (RBE) (%)	<4	<7	<10
V63 Gy (RBE) (cc)	<1	<2	<3
Bladder			
V50 Gy (RBE) (%)	<30	<32.5	<35
V60 Gy (RBE) (%)	<15	<17.5	<20
Bowel			
V50 Gy (RBE) (cc)	<0.5	<1	<1
Femoral Head			
Dmax (Gy (RBE))	<40	<42	<44

OAR, organ at risk; RBE, relative biological effectiveness; Dmax, maximum dose

This spot-scanning proton beam therapy corresponds to the pencil beam scanning (PBS) technique. Beam delivery was permitted only when the fiducial marker was within ±2 mm of the planned position [14, 16]. To ensure robustness, a 3-mm lateral margin and a range uncertainty margin (range × 3.5% + 1 mm) were applied. The range uncertainty margin refers to an additional margin in the beam direction (depth) that accounts for potential errors in the predicted proton range within the patient due to factors such as inaccuracies in converting CT Hounsfield units to stopping power ratios, anatomical heterogeneities, and beam modeling limitations. All treatments were delivered using the PROBEAT-CR system (Hitachi, Ltd., Tokyo, Japan), which incorporates a real-time image-guided gating system that continuously monitors the fiducial markers and enables beam delivery only when the target lies within a predefined positional threshold, ensuring accurate dose delivery. The typical beam delivery time was ~5 min per session.

### Clinical assessment and statistical analysis

Baseline IPSS was assessed prior to RGPT. Patients were categorized according to IPSS severity as mild (0–7 points), moderate (8–19 points), or severe (20–35 points), based on the American Urological Association classification [18]. Prostate volume was assessed from imaging acquired at the time of radiotherapy treatment planning, which was performed after the initiation of ADT when applicable. An age cutoff of 75 years was established in accordance with the Japanese healthcare system, which classifies individuals aged 75 or older as ‘late-stage elderly.’ In the univariable analysis, we evaluated several bladder dose–volume parameters, including V30Gy, V40Gy, V50Gy, and V60Gy. None showed a statistically significant association with grade ≥ 2 GU AE (odds ratios, 1.00–1.01; *P* = 0.63–0.98) (Supplementary Table S1). Based on these findings, and considering the clinical relevance of high-dose exposure in predicting late toxicity, as well as its consistency with the prescribed dose of 63 Gy, we selected V60Gy as the representative dosimetric parameter for further analysis. Associations between risk factors and the incidence of acute GU AEs were assessed using the chi-squared test, Welch’s *t*-test, Mann–Whitney *U* test, and logistic regression analysis. The following variables were included in the multivariable logistic regression analysis: age group (<75 or ≥75 years), IPSS severity, NCCN risk group, comorbidities (hypertension, diabetes), spacer placement, use of anticoagulants or urinary symptom medications, bladder V60 (%), and prostate volume (cm^3^). Statistical significance was set at *P* < 0.05. Because this was an exploratory study, we included all variables considered to have potential clinical relevance, regardless of their statistical significance in univariable analysis [19]. All statistical analyses were performed using EZR (Jichi Medical University, Tochigi, Japan), a graphical user interface for R (The R Foundation for Statistical Computing, Vienna, Austria). EZR is a modified version of R Commander that incorporates statistical functions frequently used in biostatistics [20].

## RESULTS

### Patient characteristics

The analysis included 326 patients with a median age of 72 years (range, 50–89). Based on the NCCN risk classification, 90 (27.6%), 176 (54.0%), and 60 (18.4%) patients were classified as intermediate, high, and very high risk, respectively. Based on the baseline IPSS (median: 8 [0–30] points), 150 (46.0%), 148 (45.4%), and 28 (8.6%) patients were classified as having mild, moderate, and severe symptoms, respectively. Patient characteristics are summarized in Table 2. Notable pretreatment conditions included anticoagulant medication use (*n* = 76, 23.3%), hypertension (*n* = 142, 43.6%), and diabetes mellitus (*n* = 53, 16.3%).

**Table 2:** Patient characteristics

Variables		(*n* = 326)
Age (year)		
	Median [range]	72 [50–89]
Age group, n (%)		
	<75	201 (61.7)
	≥75	125 (38.3)
NCCN risk group, n (%)		
	Low	0 (0)
	Intermediate	90 (27.6)
	High	176 (54.0)
	Very High	60 (18.4)
Clinical stage, n (%)		
	I	89 (27.3)
	II	60 (18.4)
	III	177 (54.3)
	IV	0 (0)
Baseline IPSS score		
	Median [range]	8.00 [0–30]
IPSS group, n (%)		
	Mild	150 (46.0)
	Moderate	148 (45.4)
	Severe	28 (8.6)
Spacer placement, n (%)		
	−	122 (37.4)
	+	204 (62.6)
Hypertension, n (%)		
	−	184 (56.4)
	+	142 (43.6)
Diabetes, n (%)		
	−	273 (83.7)
	+	53 (16.3)
Anticoagulant therapy, n (%)		
	−	250 (76.7)
	+	76 (23.3)
Pretreatment medication for urinary symptoms, n (%)		
	−	252 (77.3)
	+	74 (22.7)

IPSS, International Prostate Symptom Score; NCCN, National Comprehensive Cancer Network

### Findings from statistical analysis

Grade 2 acute GU AEs were observed in 127 patients (39.0%), and no grade ≥ 3 toxicity was noted in this cohort. As shown in Table 3, baseline IPSS and age were significantly associated with the development of grade ≥ 2 GU AEs. Compared with patients who experienced grade < 2 GU AEs, those with grade ≥ 2 events had a significantly higher median baseline IPSS (11.00 vs 7.00, *P* < 0.001) and median age (74 vs 72 years, *P* = 0.03). Both baseline IPSS and age were significantly associated with the occurrence of grade ≥ 2 acute GU AEs in univariate and multivariable logistic regression analyses (Table 4). On multivariable analysis, IPSS severity was significantly associated with grade ≥ 2 AEs, with odds ratios (OR) and 95% confidence intervals (CIs) of 1.71 (95% CI: 1.03–2.82) for the moderate group and 3.75 (95% CI: 1.49–9.46) for the severe group, compared with the mild group (both *P* = 0.01). Age group was also a significant factor (OR = 1.80; 95% CI: 1.10–2.95; *P* = 0.02). The discriminative performance of the multivariable logistic regression model was evaluated using the area under the receiver operating characteristic curve (AUC), which was 0.66 (95% confidence interval: 0.60–0.72). Model calibration was assessed using the Hosmer–Lemeshow goodness-of-fit test, which showed an adequate fit (chi-squared = 5.04, df = 8, *P* = 0.75). No statistically significant associations were found for other variables (e.g. clinical stage, Gleason score, NCCN risk group, anticoagulant therapy, pretreatment medication for urinary symptoms, spacer placement, diabetes, and hypertension). In addition, no significant differences were found in terms of prostate volume, bladder volume, and median bladder dose metrics from V30 to V60 (Supplementary Table S2).

**Table 3:** Predisposing factors for acute genitourinary adverse events

Patient characteristics		Acute GU AE		
		Grade < 2	Grade ≥ 2	*P* value
		(*n* = 199)	(*n* = 127)	
Age (year)				
	Median [range]	72 [50–89]	74 [56–86]	0.03
Age group, n (%)				
	<75	134 (67.3)	67 (52.8)	0.01
	≥75	65 (32.7)	60 (47.2)	
NCCN risk group, n (%)				
	Intermediate	51 (25.6)	39 (30.7)	0.28
	High	109 (54.8)	67 (52.8)	
	Very High	39 (19.6)	21 (16.5)	
Clinical stage, n (%)				
	I	54 (27.1)	35 (27.6)	0.65
	II	40 (20.1)	20 (15.7)	
	III	105 (52.8)	72 (56.7)	
Baseline IPSS score				
	Median [range]	7.00 [0–28]	11.00 [1–30]	<0.001
IPSS group, n (%)				
	Mild	104 (52.3)	46 (36.2)	<0.001
	Moderate	85 (42.7)	63 (49.6)	
	Severe	10 (5.0)	18 (14.2)	
Spacer placement, n (%)				
	−	70 (35.2)	52 (40.9)	0.35
	+	129 (64.8)	75 (59.1)	
Hypertension, n (%)				
	−	112 (56.3)	72 (56.7)	1
	+	87 (43.7)	55 (43.3)	
Diabetes, n (%)				
	−	168 (84.4)	105 (82.7)	0.79
	+	31 (15.6)	22 (17.3)	
Anticoagulant therapy, n (%)				
	−	152 (76.4)	98 (77.2)	0.98
	+	47 (23.6)	29 (22.8)	
Pretreatment medication for urinary symptoms, n (%)				
	−	155 (77.9)	97 (76.4)	0.86
	+	44 (22.1)	30 (23.6)	

GU, genitourinary; AE, adverse event; NCCN, National Comprehensive Cancer Network; IPSS, International Prostate Symptom Score

**Table 4:** Univariate and multivariable analyses of predisposing factors for acute genitourinary adverse events

Variables	Strata	Univariate logistic regression			Multivariable logistic regression		
		OR	95% CI	*P* value	OR	95% CI	*P* value
Age (year)							
	Age	1.04	1.00, 1.08	0.04			
Age Group							
	<75	reference			reference		
	≥75	1.85	1.17, 2.92	0.009	1.8	1.10, 2.95	0.02
NCCN risk group							
	Intermediate	reference			reference		
	High	0.8	0.48, 1.35		0.65	0.36, 1.19	
	Very High	0.7	0.36, 1.38	0.56	0.45	0.19, 1.03	0.16
Baseline IPSS score							
	per 1 increase	1.07	1.03, 1.10	<0.001			
IPSS group							
	Mild	reference			reference		
	Moderate	1.68	1.04, 2.70		1.71	1.03, 2.82	
	Severe	4.07	1.74, 9.50	0.002	3.75	1.49, 9.46	0.01
Prostate volume (cc)							
	per 1 cc increase	1.01	1.00, 1.03	0.08	1.02	1.00, 1.04	0.14
Bladder V60 (%)							
	per 1% increment	1	0.93, 1.08	0.98	0.93	0.84, 1.03	0.19
Spacer placement							
	No	reference			reference		
	Yes	0.783	0.50, 1.24	0.29	0.67	0.37, 1.18	0.16
Hypertension							
	No	reference			reference		
	Yes	0.983	0.63, 1.54	0.94	0.9	0.56, 1.44	0.65
Diabetes							
	No	reference			reference		
	Yes	1.14	0.62, 2.07	0.68	1.16	0.61, 2.21	0.64
Anticoagulant therapy							
	No	reference			reference		
	Yes	0.957	0.56, 1.62	0.87	0.87	0.49, 1.52	0.62
Pretreatment medication for urinary symptoms							
					
	No	reference			reference		
	Yes	1.09	0.64, 1.85	0.75	0.66	0.36, 1.20	0.17

OR, odds ratio; IPSS, International Prostate Symptom Score; GU, genitourinary; NCCN, National Comprehensive Cancer Network

## DISCUSSION

This study represents one of the largest investigations of AEs in PBT for prostate cancer. In this cohort of 326 patients, grade ≥ 2 GU AEs occurred in 39.0% of cases and were significantly associated with higher baseline IPSS and older age. Notably, the incidence is relatively higher than that reported in previous studies. Table 5 summarizes prior research on the incidence of acute GU AEs in prostate cancer patients treated with PBT [5, 6, 11, 21–25]. Reported rates of grade ≥ 2 acute GU AEs widely varied, ranging from 5% to 42%, likely reflecting differences in study populations, treatment methods, fractionation schedules, evaluation criteria, and institutional practices in reporting AEs.

**Table 5:** The incidence of acute genitourinary toxicity in patients undergoing proton beam therapy

Author	No. of patients	Delivery technique	Toxicity Grading Scale	Total dose (Gy (RBE))	Gy (RBE) /Fr	Baseline IPSS score (Mild/Mod/Sev, %)	Age (year) Median [range]	Acute GU toxicities (%)	
								Grade ≥ 2	Grade ≥ 3
Mayahara (23)	287	passive	NCI-CTC v 2.0	74	2	NR	NR	40	1
Nihei (25)	151	passive	NCI-CTC v 2.0	74	2	NR	67 [51–82]	12	0
Nakajima (11)	254	passive	CTCAE v 4.0	74–78	2	7 [0–29]^a^	70 [52–88]	15	0
	272	passive		60–63	3	6 [0–31]^a^	69 [47–86]	5.9	0
Iizumi (5)	73	passive	CTCAE v 4.0	74–78	2	45.2/50.7/4.1	69 [57–86]	16.4	1.4
	100	passive		70	2.5	46.0/52.0/2.0	67 [53–79]	11	0
	116	passive		63	3	45.7/50.0/4.3	68 [53–79]	13.8	0
Mishra (24)	1105	passive	CTCAE v 4.0	Conventional fractionation		66.0/29.2/4.7	65.2 (7.6)^b^	15	0.2
	238	scanning		Conventional fractionation		67.7/28.6/3.8	66.6 (6.4)^b^	22	0.4
Grewal (21)	184	passive/scanning	CTCAE v 4.0	70	2.5	8.1 [NR]^a^	NR [50–83]^c^	12.5	NR
Kim (22)	82	scanning	CTCAE v 3.0	35–60	3–7	NR	68 [44–85]	5	0
Takaoka (6)	120	scanning	CTCAE v 4.0	60–63	3	53.3/46.7/0	71 [49–84]	28	0
	107	scanning		76–78	2	49.2/50.8/0	73 [47–86]	42	0
Current study	326	scanning	CTCAE v 5.0	63	3	46.0/45.4/8.6	72 [50–89]	39	0

GU, genitourinary; NR, not reported; NCI-CTC, National Cancer Institute Common Toxicity Criteria; CTCAE, Common Terminology Criteria for Adverse Events; v, version

^a^Median [range]

^b^Mean (standard deviation)

^c^Total age was not reported in Grewal *et al.*; values are presented by NCCN risk group for comparison: 64 [53–75] for Low, 67 [50–80] for Favorable intermediate, and 68 [50–83] for Unfavorable intermediate groups, respectively.

Notably, no patients in our cohort experienced acute grade ≥ 3 GU toxicity, demonstrating the excellent tolerability of our PBT protocol. This compares favorably with major photon-based trials; for instance, the Conventional or Hypofractionated High-dose Intensity Modulated Radiotherapy in Prostate Cancer (CHHiP) and the Prostate Fractionated Irradiation Trial (PROFIT) trials reported acute grade ≥ 3 GU toxicity rates of ~1–2% for hypofractionated intensity-modulated radiation therapy (IMRT). Several factors likely contributed to this result. Technologically, the spot-scanning irradiation method enables precise dose painting and substantially reduces the integral dose to the bladder and urethra compared to traditional techniques. Furthermore, our clinical management focused on early pharmacological intervention. Tsirkas *et al.* reported that proactively initiating tamsulosin in patients receiving external beam radiotherapy effectively mitigates symptom severity and may prevent acute urinary retention [26]. In line with these findings, our institutional practice of proactively initiating alpha-blockers at the first sign of urinary symptoms likely played a crucial role in preventing progression to grade ≥ 3 GU events requiring invasive intervention. These results suggest that the combination of advanced proton delivery technology and vigilant early supportive care can effectively prevent severe acute GU complications. Furthermore, the inherent structure of the Common Terminology Criteria for Adverse Events (CTCAE) grading system should be considered when interpreting these results. Under CTCAE v5.0, common acute symptoms such as urinary frequency and urgency are categorized only up to grade 2, even if clinically severe. In contrast, the Radiation Therapy Oncology Group (RTOG) acute radiation morbidity scoring criteria allow for grade 3 classification for extreme frequency, nocturia, severe dysuria, pelvic pain, or bladder spasms requiring regular narcotic analgesics. Therefore, the zero incidence of grade 3 events in our study partly reflects the fact that even clinically significant increases in urinary frequency or urgency are capped at grade 2 within the CTCAE system, regardless of their intensity.

Takaoka *et al.* examined 227 patients treated with PBS and compared patients who underwent conventional fractionation (76–78 Gy [RBE] in 38–39 fractions, *n* = 107) with moderately hypofractionated PBT (60–63 Gy [RBE] in 20–21 fractions, *n* = 120) [6], revealing a significantly higher rate of grade 2 acute GU AEs in the former (42% vs 28%, *P* = 0.02). In their study, the delivery method of PBT may have influenced the variability in incidence.

In the Proton Collaborative Group (PCG) 001–09 prospective registry trial, higher rates of grade 2 acute GU adverse events were observed in patients treated with PBS compared to those who underwent passive scattering/uniform scanning PBT (PS/US PBT). PBT-related toxicity can be influenced by any treatment- and planning-specific variables. Proton dose distribution is highly sensitive to anatomical changes and motion-related factors, which can lead to discrepancies between the planned and actually delivered doses. These effects are more pronounced with PBS than with PS/US PBT. Similarly, linear energy transfer distributions within the target are highly inhomogeneous in PBS, and the most heavily weighted spots can be located near or within adjacent normal tissues, potentially increasing the RBE-weighted dose in these regions [27]. Similarly, Nakajima *et al.* reported that univariate analysis revealed that PBS was significantly associated with more frequent grade 2 acute GU toxicity than PS [11]. Further studies are warranted to fully understand the influence of the delivery method on GU toxicities since PBS is becoming increasingly adopted worldwide.

Another possible contributor to the variability in the reported AE rates may be the difference in institutional supportive care practices following the initiation of radiation therapy. This is particularly relevant considering that toxicity assessment is, to some extent, subject to physician interpretation. At our institution, alpha-1 blockers are proactively offered to patients with mild to moderate urinary symptoms after the initiation of RGPT, based on shared decision-making. Although this is an inherent limitation to any study using clinician-scored toxicities as a study end point, the differences in posttreatment supportive care for urinary symptoms could strongly contribute to the observed discrepancies in reported GU AE rates.

As noted above, the frequency of acute GU AEs can vary greatly between studies due to multiple factors. Since analyses of the factors associated with acute GU AEs are based on these observed frequencies, they are, in principle, even more susceptible to interstudy variability. Therefore, the most reliable determinants are those consistently reported across multiple studies, demonstrating reproducibility. Among the potential predictors, baseline IPSS is a strong candidate. The IPSS is a widely accepted and validated metric for urinary function, and multiple studies have identified it as a significant predictor of acute GU AEs [9–12, 24]. For example, Hasan *et al.* identified baseline IPSS, age, nodal irradiation, and treatment site as independent predictors of acute GU toxicity [9], although their cohort included a high proportion of patients receiving nodal irradiation and conventional fractionation, limiting direct comparability with our study. Meanwhile, Nakajima *et al.* reported that baseline IPSS significantly predicted acute GU AEs in a cohort of 526 patients with conventionally fractionated (74–78 Gy [RBE] in 37–39 fractions) or hypofractionated (60–63 Gy [RBE] in 20–21 fractions) PBT [11]. Similarly, Zhang *et al.* and Malik *et al.* observed a significant correlation between higher baseline IPSS scores and acute GU toxicities, albeit in a cohort that underwent treatment modalities other than PBT [10, 12].

Old age is also a frequently reported independent predictor of grade ≥ 2 acute GU toxicity. Mayahara *et al.* identified age as a significant factor in univariate analysis [23], while Hasan *et al.* confirmed its significance in multivariable analysis (HR = 1.04, 95% CI: 1.006–1.07, *P* = 0.046) [9]. However, age was not a significant predictor in other studies [6, 11]. Despite these discrepancies, older patients may be more prone to an increased risk of acute GU AEs due to age-related changes in lower urinary tract function, which can increase susceptibility to radiation-induced damage [28].

Unexpectedly, bladder dose-volume histogram (DVH) parameters were not significant predictors of acute GU toxicity in our study. A primary explanation is the remarkable dose reduction achieved with PBT. In our cohort, median bladder doses in both the <G2 and ≥ G2 groups were roughly V30 = 20%, V40 = 15%, V50 = 10%, and V60 = 5%. These values are considerably lower than the dose–volume constraints typically utilized in photon-based radiotherapy. When doses are consistently maintained below such low thresholds in all patients, a clear dose–response relationship is often difficult to detect.

This is supported by Kubo *et al.*, who found that bladder V5–V60 were not associated with toxicity in a hypofractionated IMRT cohort where doses were well-managed [29]. These findings suggest that in the context of high-precision therapy like PBT, the physical dosimetric parameters to the whole bladder may no longer be the primary limiting factor for acute toxicity. Instead, attention may need to shift toward individual biological sensitivity or other localized dosimetric parameters, such as the dose to the prostatic urethra or bladder trigone, which were not specifically evaluated in this study. Furthermore, future comparative studies between proton and photon therapy using identical dose-fractionation schedules are warranted to clarify the distinct roles of these dosimetric and biological factors.

In this study, baseline IPSS severity and age were identified as independent significant predictors of acute grade ≥ 2 GU adverse events. The multivariable model incorporating these variables demonstrated good calibration (Hosmer–Lemeshow test *P* = 0.75), thereby supporting their clinical utility for risk stratification. However, the model’s discriminative performance was limited (AUC 0.66; 95% CI, 0.60–0.72). While clinical factors such as age and IPSS are significant, their predictive power is limited in the absence of detailed dosimetric data. In particular, the radiation dose delivered to the prostatic urethra and bladder neck is a critical determinant of acute GU toxicity, but these structures were not separately contoured in our study and therefore could not be incorporated into our multivariable model. This omission likely accounts for a substantial portion of the unexplained variance. Furthermore, as supported by recent literature, individual biological heterogeneity and baseline comorbidities may contribute to divergent outcomes. Kerns *et al.* emphasized that radiotherapy adverse effects cannot be accounted for by clinical and dosimetric factors alone, highlighting the influence of genetic polymorphisms in DNA repair pathways [30]. Similarly, Stanić *et al.* noted that standard protocols often overlook individual differences in radiosensitivity and the role of proinflammatory cytokines [31]. Underlying conditions affecting vascular or neurological bladder function may further confound individual risk. Therefore, we propose that our model is best utilized as a population-level screening tool rather than for definitive individual outcome prediction. By identifying cohorts at a higher relative risk, clinicians can implement more vigilant monitoring and earlier supportive care, providing a pragmatic framework for toxicity management in standard clinical workflows, where high-resolution dosimetric or biological data are not routinely available. The absence of significant associations with other conventional clinical factors, such as DVH parameters or comorbidities, suggests that IPSS and age alone are insufficient to accurately discriminate events at the individual-patient level. Thus, although the model appears appropriate for estimating risk at the population level, further exploration of additional, previously unassessed predictors—such as genetic predispositions or more detailed biological markers—is warranted to achieve more precise individualized prediction.

The strengths of this study include its relatively large sample size and use of prospective cohort data. However, several limitations must be acknowledged. First, a single-center design may introduce institutional bias. Second, potential variability among individual physicians in evaluating acute GU AEs may influence the consistency of findings. Third, since the data were collected under specific institutional practices, the prognostic factors identified may not be fully generalizable. Another significant limitation is the absence of longitudinal patient-reported outcomes (PROs). While baseline IPSS was collected, serial posttreatment PRO data were not routinely obtained. This study focused on predictors of physician-reported CTCAE grading; however, physician-graded toxicity and patient-reported symptom severity often diverge, with clinicians frequently underestimating patients’ symptom burden [32, 33]. The lack of longitudinal IPSS data prevents a full trajectory of urinary symptom deterioration from the patient’s perspective. Future prospective studies should incorporate both physician- and patient-reported endpoints to provide a more comprehensive assessment of acute toxicity and identify predictors that are meaningful for both healthcare providers and patients. Nevertheless, our findings confirm that higher baseline IPSS and older age are robust and reproducible predictors of acute GU toxicity. Thus, assessing IPSS before treatment can help clinicians optimize treatment plans and supportive care strategies to mitigate the risk of GU AEs in patients undergoing PBT for prostate cancer.

Among patients with localized prostate cancer treated with RGPT, those with moderate-to-severe baseline IPSS and older age were at significantly higher risk of developing grade ≥ 2 toxicity. These findings underscore the clinical relevance of pretreatment IPSS assessment, highlighting its potential utility in guiding individualized treatment plans and supportive care strategies.

## Supplementary Material

Supplementary_material_rrag012

## Data Availability

The datasets generated and/or analyzed during the current study are not publicly available due to institutional policy and concerns regarding participant confidentiality, but are available from the corresponding author on reasonable request.
